# Physician experiences with and perceptions of risk evaluation and mitigation strategy programs with elements to assure safe use

**DOI:** 10.1371/journal.pone.0288008

**Published:** 2023-07-06

**Authors:** Ameet Sarpatwari, Beatrice L. Brown, Sarah A. McGraw, Sara Z. Dejene, Abdurrahman Abdurrob, Aaron S. Kesselheim

**Affiliations:** 1 Program On Regulation, Therapeutics, And Law (PORTAL), Division of Pharmacoepidemiology and Pharmacoeconomics, Department of Medicine, Brigham and Women’s Hospital and Harvard Medical School, Boston, Massachusetts, United States of America; 2 The Hastings Center, Garrison, New York, United States of America; World Health Organization, BELGIUM

## Abstract

**Purpose:**

The US Food and Drug Administration (FDA) Amendments Act of 2007 authorized the FDA to require risk evaluation and mitigation strategy (REMS) programs for drugs with important safety concerns. REMS can have elements to assure safe use (ETASU), such as patient registries, dispensing restrictions, and physician training and certification requirements. We aimed to understand physician experiences with and perceptions of a selection of ETASU REMS.

**Methods:**

Physicians prescribing 1 of 4 ETASU REMS-covered drugs: natalizumab, riociguat, sodium oxybate, and vigabatrin.

**Study design:**

Descriptive phenomenological study based on semi-structured phone interviews.

**Data collection/Extraction methods:**

Qualitative content analysis to summarize physician responses to open-ended questions.

**Results:**

Of 31 physicians (14 female), 6 prescribed riociguat, 6 vigabatrin, 7 sodium oxybate, and 12 natalizumab (5 for Crohn’s disease, 7 for multiple sclerosis), most demonstrated good understanding of the rationale for and requirements of the ETASU REMS but believed that the programs had limited effect on clinical practice. Some physicians reported that the ETASU REMS made them more comfortable with prescribing covered drugs due to heightened oversight, facilitated discussions about treatment, and were likely more beneficial for non-specialists. Concerns were raised about the administrative effort needed to comply with the programs and the potential misuse of patient health information transmitted to manufacturers.

**Conclusions:**

Physicians are generally aware of ETASU REMS and get reassurance from the additional oversight, but the programs can be better integrated into clinical workflows and can be designed to better protect patient health information.

## 1. Introduction

The Food and Drug Administration (FDA) approves new prescription drugs if the benefits appear to outweigh the risks. However, some new drugs have severe risks for certain populations, such as pregnant women [1, 2], while other new drugs have risks that can be reduced with better surveillance, such as regular lab tests [3, 4]. To ensure that these drugs can still be FDA-approved and made available for the patients who might benefit from them, Congress gave FDA the authority to require manufacturers to set up risk evaluation and mitigation strategy (REMS) programs [5]. Some REMS programs consist of a medication guide and a communication plan to instruct patients and physicians on safe drug use. Others include additional elements to assure safe use (ETASU), which can include patient registries, dispensing restrictions such as requiring evidence that a patient is taking birth control or has received the appropriate screening blood tests, and training and certification of prescribing physicians and dispensing pharmacies [6]. As of May 24, 2023, 63 FDA-approved drugs or drug classes had REMS programs, of which 59 (94%) had ETASU, while only 2 (3%) had a medication guide and/or a communication guide alone [7].

Despite the prevalence of ETASU REMS, in a 2013 report, the US Department of Health and Human Services Office of the Inspector General cast doubt on their effectiveness [8]. The evidence to date has been mixed. One review of extended-release/long-acting opioids found that between 2012 and 2016 only 28% of prescribers had taken the continuing education tied to the drugs’ ETASU REMS [9]. However, a different study found a lower incidence of opioid overdose among Medicaid beneficiaries following the program’s implementation (which the authors acknowledge could not be disentangled “from many other ongoing initiatives with similar goals”) [10].

Varied findings have also been reported for the impact of ETASU REMS on physician prescribing practices. An investigation of the thrombopoietin-agonist eltrombopag (Promacta) observed decreased off-label use while the drug was covered, although that indication was later FDA-approved [11]. A study of transmucosal immediate-release fentanyl (TIRF) drugs that were supposed to be restricted to opioid-tolerant patients found that by 60-months post ETASU REMS implementation, 35%-55% of patients met this criterion and 34% of surveyed prescribers had prescribed these drugs inappropriately [12]. However, a separate investigation of Medicare Part D beneficiaries reported that between 2010 and 2014, the TIRF ETASU REMS was associated with a sustained decrease in the percent of prescriptions among patients without known opioid tolerance [13]. Finally, a study of the ETASU REMS for erythropoiesis stimulating agents darbepoetin alfa and epoetin alfa did not find a change in non-evidence-based initiation of either drug following initiation of the program [14].

Given the uncertainty over the impact of these programs, we sought to better understand physicians’ experiences with and perceptions of the operation of ETASU REMS in clinical practice.

## 2. Methods

We conducted a descriptive phenomenological study, using semi-structured guides to interview a cohort of physicians who prescribed ETASU-REMS covered drugs, and qualitatively analyzed their perceptions and experiences to learn how these programs can be better designed and implemented.

To select the programs being examined, we ranked all 40 ETASU REMS existing as of January 2016 on the rigor of their requirements, including: (1) prescriber certification (score 0 if no, 1 if yes), (2) monitoring or testing (0 if no, 1 if recommended, 2 if mandated, 3 if data reporting requirements), (3) administration or dispensing restrictions (0 if no, 1 if certification required, 2 if only limited specialty pharmacies or centers could qualify), and (4) manufacturer access to patient health information (0 if no, 1 if yes). We selected four with scores higher than the median: natalizumab (Tysabri), riociguat (Adempas), sodium oxybate (Xyrem), and vigabatrin (Sabril). Table 1 provides details on the indications and risks of these drugs, and on their monitoring and testing requirements.

**Table 1:** ETASU REMS-covered study drugs. ETASU = elements to assure safe use, PML = progressive multifocal leukoencephalopathy, REMS = risk evaluation and mitigation strategy, * = Removed in June 2016.

Drug	Indication	Risk Prompting ETASU REMS	Screening/Monitoring or Testing Requirements	Dispensing/Administration Limitations	Data Sharing Statement in Patient Enrollment Agreement
Natalizumab (Tysabri)	Crohn’s disease, multiple sclerosis	PML, an opportunistic, often fatal infection caused by the JC virus, the estimated incidence of which is 11/1,000 among individuals with: (1) presence of anti-JC virus antibodies, (2) prior immunosuppressant use, and (3) long-term (>2 years) natalizumab use	Recommended monitoring for PML at 3 and 6 months and every 6 months thereafter; mandatory submission of discontinuation monitoring form	Certified infusion centers	“I understand that by signing this Authorization, I authorize my prescriber, infusion site, pharmacy, and/or health insurance company to disclose the PHI in my medical records to Biogen Idec Inc. and its representatives or agents, including information related to my medical condition, treatment, and health insurance, as well as all information provided on any prescription.”
Riociguat (Adempas)	Chronic thromboembolic pulmonary hypertension, pulmonary arterial hypertension	Birth defects, including cardiac ventricular-septal defect; maternal toxicity; and spontaneous abortions	Females of reproductive potential: mandatory baseline and monthly pregnancy tests	Certified pharmacies	N/A
Sodium oxybate (Xyrem)	Cataplexy or excessive daytime sleepiness in narcolepsy	Central nervous system and respiratory depression, associated with concomitant use of sedative hypnotics or alcohol	Screening for substance abuse; mandatory monitoring at 3 months	Centralized pharmacy (mail only)	N/A
Vigabatrin (Sabril)	Refractory complex partial seizures (>10 years), infantile spasms (<2 years)	Mild-to-severe vision loss in 30 percent or more of patients associated with increasing dose and cumulative exposure	Mandatory vision testing every 90 days*	Certified pharmacies	“I authorize my healthcare providers and health plans to disclose personal and medical information related to my use or potential use of Sabril (vigabatrin) to Lundbeck and its agents and contractors and I authorize Lundbeck to use and disclose this information to: 1) establish my benefit eligibility; 2) communicate with my healthcare providers and health plans about my benefit and coverage status and my medical care; 3) provide support services, including facilitating the provision of Sabril to me; 4) evaluate the effectiveness of Sabril’s education programs; and 5) participate in the Sabril Patient Registry.”

### 2.1. Interview guide, physician recruitment, and conduct of interviews

We developed a semi-structured interview guide with three primary topics: utility and burden; privacy; and recommended improvements (S1 Appendix in S1 File). To minimize bias, we posed open-ended questions. We tested the guide in pilot interviews and made minor revisions to it for clarity based on our experiences.

Eligible participants must have prescribed the drug of interest within two years of their interview. To recruit participants, we identified physicians associated with national patient support groups for diseases treated by the drugs. Aiming to assemble a diverse group, we intentionally sought out physicians from states across the country. Invitation emails to these physicians included a fact sheet providing an overview of the study and its goals.

Interviews were conducted between 2016 and 2017 by two research assistants, AA (male, BA) and SZD (female, BA), who were trained through practice interviews led by AS (male, PhD/JD). In each interview, the interviewer noted his or her background and spoke alone with the participant. Interviews were approximately one hour, conducted over the phone, recorded, and transcribed. Field notes were taken as needed. Owing to resource constraints, repeat interviews were not conducted, nor were transcripts returned to participants for comment or correction. Participants completing an interview received a $150 gift card honorarium. A target of at least 5 interviews per drug were sought, with interviews stopped once data saturation was reached.

### 2.2. Content analysis

Reporting of study methods and results adhered to Consolidated Criteria for Reporting Qualitative Research guidelines [15]. We employed qualitative content analysis to summarize physician responses to the open-ended questions [16, 17]. These responses can be coded using deductive categories defined *a priori* as well as categories derived inductively, based on concepts newly identified through constant comparison or by line-by-line reading of the interview text [18]. After reading the transcripts, the research team generated a consensus start list of codes. One person (SAM) then coded each transcript using NVivo, meeting with other members of the team to review and refine the list and code definitions (S2 Appendix in S1 File). Finally, the research team reviewed the data to identify themes within and across codes. Again, owing to resource constraints, participant feedback on findings was not sought. This study was approved by the Brigham and Women’s Hospital Institutional Review Board.

## 3. Results

Among 31 physician participants, 6 prescribed riociguat, 6 vigabatrin, and 7 sodium oxybate (Table 2). Five physicians prescribed natalizumab for Crohn’s disease and 7 for multiple sclerosis. Fourteen were female, 13 practiced in Massachusetts, and 28 worked at academic medical centers.

**Table 2:** Characteristics of participating physicians.

Physician Number	Drug	Sex	Specialty	Academic Medical Center	State
1	riociguat (Adempas)	Female	Pulmonologist	Yes	MA
2	riociguat (Adempas)	Female	Cardiologist	Yes	CT
3	riociguat (Adempas)	Female	Pulmonologist	Yes	RI
4	riociguat (Adempas)	Male	Pulmonologist	Yes	NY
5	riociguat (Adempas)	Male	Pulmonologist	Yes	CA
6	riociguat (Adempas)	Male	Cardiologist	Yes	MN
7	vigabatrin (Sabril)	Male	Pediatric Neurologist	Yes	WA
9	vigabatrin (Sabril)	Female	Pediatric Neurologist	Yes	CA
8	vigabatrin (Sabril)	Male	Pediatric Neurologist	Yes	CA
10	vigabatrin (Sabril)	Male	Pediatric Neurologist	Yes	OH
11	vigabatrin (Sabril)	Female	Pediatric Neurologist	Yes	NY
12	vigabatrin (Sabril)	Female	Pediatric Neurologist	Yes	AL
13	natalizumab (Tysabri)-Crohn’s	Male	Gastroenterologist	Yes	MA
14	natalizumab (Tysabri)-Crohn’s	Female	Gastroenterologist	No	RI
15	natalizumab (Tysabri)-Crohn’s	Male	Gastroenterologist	Yes	MA
16	natalizumab (Tysabri)-Crohn’s	Male	Gastroenterologist	Yes	MA
17	natalizumab (Tysabri)-Crohn’s	Female	Pediatric Gastroenterologist	Yes	CA
18	natalizumab (Tysabri)-Multiple Sclerosis	Female	Neurologist	Yes	MA
19	natalizumab (Tysabri)-Multiple Sclerosis	Male	Neurologist	Yes	MA
20	natalizumab (Tysabri)-Multiple Sclerosis	Male	Neurologist	Yes	MA
21	natalizumab (Tysabri)-Multiple Sclerosis	Male	Neurologist	No	RI
22	natalizumab (Tysabri)-Multiple Sclerosis	Male	Neurologist	Yes	MA
23	natalizumab (Tysabri)-Multiple Sclerosis	Female	Neurologist	Yes	MA
24	natalizumab (Tysabri)-Multiple Sclerosis	Female	Neurologist	Yes	MA
25	sodium oxybate (Xyrem)	Female	Neurologist	Yes	MA
26	sodium oxybate (Xyrem)	Male	Neurologist	Yes	MA
27	sodium oxybate (Xyrem)	Male	Pediatric Neurologist	No	FL
28	sodium oxybate (Xyrem)	Female	Pulmonologist	Yes	MI
29	sodium oxybate (Xyrem)	Male	Pulmonologist	Yes	MA
30	sodium oxybate (Xyrem)	Male	Neurology	Yes	GA
31	sodium oxybate (Xyrem)	Female	Psychiatrist	Yes	AZ

### 3.1. Knowledge of ETASU REMS

Participants demonstrated good knowledge of the ETASU REMS. Most understood why the FDA required the programs for the drugs that they prescribed. For example, one participant (physician 13, natalizumab) explained, “My understanding of this program is that it was mandated by the FDA for the rerelease of the drug,” which had been removed owing to the risk of progressive multifocal leukoencephalopathy, “to limit potential, maybe to limit irresponsible or inappropriate use of the agent.” Most participants also knew the requirements of the programs (Table 3). Summarizing the ETASU REMS for vigabatrin, one participant (physician 7) stated, “Most physicians…have to register is one component of it. Then patients…have to fill out recognition or information about the potential risk of the medication. Plus, they have to have regular examinations before treatment by ophthalmologists to exclude one of the significant potential side effects of the renal toxicity.”

**Table 3:** Selected participant comments on ETASU REMS: Awareness of requirements and impact on practice.

Theme	Response
Awareness of Requirements	Natalizumab (physician 20) There’s a form that we fill out, every-6-months sort of thing. Whether or not the patient is JC positive versus negative or whether or not they’ve been on steroids more recently. Whether or not they’ve been on any immunosuppressant medications. So, we have to submit that and update their certification for the drug. Riociguat (physician 5) But in the big view of the picture, we do mandate that our patients have regular pregnancy testing. Sodium oxybate (physician 25) First of all, even before [the drug] is given the patient, both the physician and the pharmacist would go over safety issues, particularly the excessive sedation and possibility of the drug taking effect relatively quickly…Of course, the patient has to be monitored for any side effects. I see the patient regularly and follow up depending on the intensity of their disease. I would see them every three to six months, depending on how they do. Vigabatrin (physician 8) There’s no longer the requirement to get the every-3-month visual test. My understanding prior to that was that if we filled out the form that the patient did have a neurologic condition that permitted an ophthalmologic evaluation, that we were required to do so and do so every 3 months.
Impact on Practice	*Actions* Natalizumab (phyician 20) My view is that the safety program doesn’t really affect things. What is more material is the actual side effects of the drug which need to be taken into consideration. As we’ve gained information about that, you sort of gauge risk around that versus benefit. Riociguat (physician 5) I think the reality of it is that in practice, at least in our practice, the conversation around the safety signal and understanding what the issue around safety is, takes more of a frontline decision-making role rather than the existence of the REMS program. Sodium oxybate (physician 31) I don’t start with prescribing sodium oxybate. It’s a medication that I use for specific patient reasons. So, if those patient indicators are present, it doesn’t really matter to me. I mean it takes more time, but it won’t deter me from using a medicine when I think it’s the best medicine for a patient. I’ll just do what needs to be done to prescribe it. Vigabatrin (physician 11) I think it’s because of how refractory and how ill my patients are that it doesn’t give me as much pause. *Benefits* Natalizumab (physician 17) But I think it’s nice for the physician and the patient to know that there’s another layer of checking-the-box and making sure we’re doing everything we can. Double-checking to make sure the patient is not experiencing any of those negative early signs. Riociguat (physician 6) The REMS program provides attention. It gets our attention. It gets the patient’s attention. It requires, it has a temporal reinforcement issue with it. In other words, you have to do it every month and check on it. We probably would be a little slacker than that. Sodium oxybate (physician 28) I don’t feel like I’m hanging out on a limb without any backup. And I know if there’s a problem, I have someone to contact…I think it’s as regulated as it should be… And I think patients have a sense of security that’s in place. Vigabatrin (physician 11) I think it’s a contract in a sense…We would be doing it clinically anyway if the REMS program didn’t exist, I think, but it ensures that things will happen. *Burdens* Natalizumab I think for us it’s superfluous. I think it’s just extra work that doesn’t really give much benefit. (physician 20) I think it probably just adds paperwork for me. (physician 23) Riociguat It’s not any more burden than doing the prior authorization for the medication. (physician 2) I have enormous staffing support, which doesn’t make this any less of a pain in the where else. (physician 6) Sodium oxybate It’s a bother, and I would like to think that a good doctor knows the drug well and will be prescribing it in a thoughtful fashion, and I find the degree of oversight a little bit of a nuisance. (physician 26) I mean it has made the prescription process slower, not to mention we’ve run into problems. (physician 25) Vigabatrin Whereas this drug, because having to go through the other program and the extra added burden of filling out these forms, it really doubles the work not only by the provider prescribing it, but also other personnel that have to work with the forms and then work with the family. (physician 7) I think it’s just about the volume of paperwork that ends up going in on someone with Sabril over the course of their treatment, it’s a lot. I’m grateful that I have a staff that helps with it. But if I didn’t, I think it would be onerous. (physician 11)

### 3.2. Impact on practice

The dominant perspective was that the ETASU REMS had limited impact on clinical practice (Table 3). Reasons for this perspective included that participants were already familiar with the benefits and risks of the drug and believed that requirements for educating, consenting, and monitoring or testing patients were steps they would have taken anyway. One participant (physician 27, sodium oxybate) estimated the effect on his willingness to prescribe at “zero percent,” while another (physician 3, riociguat) said “that’s very much a standard list of things we go through.” Another participant (physician 26, sodium oxybate) noted, “I have to say there is nothing that has really popped up in the Xyrem REMS Program where I was like, ‘Wow, I would have missed that.’”

Participants highlighted benefits, such as extra regulatory oversight, which made some more willing to prescribe the covered drugs. One participant (physician 5, riociguat) explained that without its monitoring system and central pharmacy, “the onus would be upon me and my team to have a more active role” Another participant (physician 30, sodium oxybate) thought the ETASU REMS encouraged more frequent formalized discussions with patients: “I’m more often having discussions, particularly…about alcohol…I’m doing it in a formal way rather than saying it once and assuming that nothing changes and everybody remembers it.” A second participant (physician 17, natalizumab) commented that this discussion was better “for the patient and the patient-physician relationship.” A few participants felt that the ETASU REMS requirements presented minimal hassle. One participant (physician 24, natalizumab) said, “It virtually takes 60 seconds to do the forms,” while another commented that the requirements, though time-consuming, were reasonable.

Several participants, however, questioned whether the ETASU REMS were worthwhile. Some were skeptical that the programs protected safety. One participant (physician 21, natalizumab) observed that although the ETASU REMS required documentation of the completion of monitoring tests, the program did not ensure that the testing was done correctly or that the results were accurate. Many participants also believed that the ETASU REMS imposed burdens on their practice from increased paperwork, patient monitoring or testing, and communication between the practice, pharmacies, and patients. Some participants were bothered by needing to respond to queries about adverse events from the sponsor. One participant (physician 27, sodium oxybate) called such requests a “nuisance,” because the manufacturer asked the prescriber to fill out “more than a page” of paperwork and “classified everything as an adverse event.” Some participants expressed frustration over the additional work required to submit prescription orders when the ETASU REMS did not link to electronic health record systems.

### 3.3. Specialists vs. non-specialists

Several participants believed that the ETASU REMS were particularly beneficial for physicians who did not have extensive experience treating the disease for which the covered drug was indicated (Table 4). As one participant (physician 5, riociguat) commented, “Ultimately, I do worry that certain caveats of safety can be missed without a REMS program in a community setting where the practitioner only sees 1 or 2 patients with pulmonary hypertension either per-year or average.”

**Table 4:** Selected participant comments on ETASU REMS: Utility for specialists vs. non-specialists and data collection.

Theme	Response
Awareness of Requirements	Natalizumab (physician 20) There’s a form that we fill out, every-6-months sort of thing. Whether or not the patient is JC positive versus negative or whether or not they’ve been on steroids more recently. Whether or not they’ve been on any immunosuppressant medications. So, we have to submit that and update their certification for the drug. Riociguat (physician 5) But in the big view of the picture, we do mandate that our patients have regular pregnancy testing. Sodium oxybate (physician 25) First of all, even before [the drug] is given the patient, both the physician and the pharmacist would go over safety issues, particularly the excessive sedation and possibility of the drug taking effect relatively quickly…Of course, the patient has to be monitored for any side effects. I see the patient regularly and follow up depending on the intensity of their disease. I would see them every three to six months, depending on how they do. Vigabatrin (physician 8) There’s no longer the requirement to get the every-3-month visual test. My understanding prior to that was that if we filled out the form that the patient did have a neurologic condition that permitted an ophthalmologic evaluation, that we were required to do so and do so every 3 months.
Impact on Practice	*Actions* Natalizumab (physician 20) My view is that the safety program doesn’t really affect things. What is more material is the actual side effects of the drug which need to be taken into consideration. As we’ve gained information about that, you sort of gauge risk around that versus benefit. Riociguat (physician 5) I think the reality of it is that in practice, at least in our practice, the conversation around the safety signal and understanding what the issue around safety is, takes more of a frontline decision-making role rather than the existence of the REMS program. Sodium oxybate (physician 31) I don’t start with prescribing sodium oxybate. It’s a medication that I use for specific patient reasons. So, if those patient indicators are present, it doesn’t really matter to me. I mean it takes more time, but it won’t deter me from using a medicine when I think it’s the best medicine for a patient. I’ll just do what needs to be done to prescribe it. Vigabatrin (physician 11) I think it’s because of how refractory and how ill my patients are that it doesn’t give me as much pause. *Benefits* Natalizumab (physician 17) But I think it’s nice for the physician and the patient to know that there’s another layer of checking-the-box and making sure we’re doing everything we can. Double-checking to make sure the patient is not experiencing any of those negative early signs. Riociguat (physician 6) The REMS program provides attention. It gets our attention. It gets the patient’s attention. It requires, it has a temporal reinforcement issue with it. In other words, you have to do it every month and check on it. We probably would be a little slacker than that. Sodium oxybate (physician 28) I don’t feel like I’m hanging out on a limb without any backup. And I know if there’s a problem, I have someone to contact…I think it’s as regulated as it should be… And I think patients have a sense of security that’s in place. Vigabatrin (physician 11) I think it’s a contract in a sense…We would be doing it clinically anyway if the REMS program didn’t exist, I think, but it ensures that things will happen. *Burdens* Natalizumab I think for us it’s superfluous. I think it’s just extra work that doesn’t really give much benefit. (physician 20) I think it probably just adds paperwork for me. (physician 23) Riociguat It’s not any more burden than doing the prior authorization for the medication. (physician 2) I have enormous staffing support, which doesn’t make this any less of a pain in the where else. (physician 6) Sodium oxybate It’s a bother, and I would like to think that a good doctor knows the drug well and will be prescribing it in a thoughtful fashion, and I find the degree of oversight a little bit of a nuisance. (physician 26) I mean it has made the prescription process slower, not to mention we’ve run into problems. (physician 25) Vigabatrin Whereas this drug, because having to go through the other program and the extra added burden of filling out these forms, it really doubles the work not only by the provider prescribing it, but also other personnel that have to work with the forms and then work with the family. (physician 7) I think it’s just about the volume of paperwork that ends up going in on someone with Sabril over the course of their treatment, it’s a lot. I’m grateful that I have a staff that helps with it. But if I didn’t, I think it would be onerous. (physician 11)
Utility for Specialists vs. Non-Specialists	Natalizumab (physician 22) I think…the REMS program really helps somebody who is not completely focused on [multiple sclerosis] to get access to that practical hands-on information. It increases the patient’s chances of getting access to this medicine. Riociguat (physician 5) Ultimately, I do worry that certain caveats of safety can be missed without a REMS program in a community setting where the practitioner only sees 1 or 2 patients with pulmonary hypertension either per-year or average. Sodium oxybate (physician 26) The level of education that’s required to use this drug and the hassle of actually prescribing it are such that there’s a number of sleep doctors who are very reluctant to prescribe it…and that might be a good thing. Vigabatrin (physician 7) Question: do you get the sense that because this drug has a REMS program it impacts other physicians’ willingness to prescribe this drug or not? Answer: Certainly, the non-specialists.
Data Collection	Natalizumab It’s basically you’re feeding their data-gathering which they’re using for their own benefit. To me that’s a disgusting invasion of privacy. It’s far too all-encompassing, but I can’t do anything about it. (physician 18) I think it’s fine if they collect the data as long as they share it…And the timely reporting of the information, because it’s fine to report it, but it’s more important to report it when it’s clinically relevant versus…a year and a month afterwards. (physician 23) Riociguat I mean it’s the privacy of the patient. I think we’re intruding enough by showing a lot to the insurance companies. But you show information to the pharma companies, I don’t think it’s their job, and they shouldn’t know. (physician 1) I can understand giving a limited amount of information…for a REMS program, but…I wouldn’t say it’s appropriate to give Social Security number and other medical history that’s not pertinent to the REMS program. (physician 4) Sodium oxybate For instance, it seems to me reasonable…for a REMS program to enquire about things that are clearly relevant based on their understanding of the drug…but having carte blanche access where they know about a patient’s depression or AIDS or whatever it might be with more delicate things that’s unnecessary sharing of information. (physician 26) In general, I think there should be an option to opt out. So that you can say “This information is made available to the pharmaceutical company. Do you agree or not?” If you don’t agree, then they don’t get it. (physician 31) Vigabatrin I think it would have to be identified what information do they need? I think also what would they do with that information? I think it would be really important to restrict what’s needed and would be used for some other purpose. (physician 7) I think as long as it’s related to this program only, that’s fine. I think the companies are good about making sure that these are separate from any of their marketing data and only specific personnel have access to it. (physician 10)

Yet some participants reported that the ETASU REMS drove patients to specialists. One participant (physician 13, natalizumab) explained: “I think that if you’re an average, practicing gastroenterologist without an interest in ulcerative colitis or Crohn’s disease…the requirement may make this a non-starter and would say, ‘I’m going to send you to the university for evaluation.’” This participant also noted that the majority of his patients were referred by another gastroenterologist. Other participants commented that the additional work associated with the ETASU REMS may be too burdensome for community physicians.

### 3.4. Data collection

Some participants identified potential benefits of program-related data collection to the field and their practice (Table 4). For example, one participant (physician 23, natalizumab) noted that mandatory data collection meant that the denominator was known. However, many participants expressed some unease about a requirement for patients to grant pharmaceutical manufacturers access to personal health information, which was part of the enrollment forms for natalizumab and vigabatrin. First, some participants argued that pharmaceutical manufacturers were not involved in patient care and that their use of such data would likely not benefit patients. Second, concerns were raised about potential breaches of confidentiality, though one participant (physician 24, natalizumab) noted that since the precedent for data collection by third parties had already been set by insurers, “whether or not the manufacturer gets this information doesn’t really matter.” Finally, some participants recognized the need for limited data collection but were concerned that the patient release statements were unnecessarily broad. Underlying such views was the perception that pharmaceutical companies were using the data for marketing. For example, one participant (physician 6, riociguat) called required data sharing a “business strategy.”

## 4. Discussion

The rationale for and requirement of the ETASU REMS were well known by physicians prescribing 4 drugs subject to these requirements. Some physicians reported greater comfort prescribing covered drugs on account of this oversight, and some believed the programs promoted physician-patient discussion. However, concerns were raised about the administration time required for compliance and potential improper use of patient health information.

Although their primary purpose is to reduce drug risks, ETASU REMS may have other benefits. By promoting better communication with patients, these programs may help strengthen the physician-patient relationship, increasing patient trust and shared decision making. There is some support for this conclusion in the medical literature. In a survey of Kaiser Permanente Southern California health care providers who had prescribed 1 of 21 REMS-covered drugs between January and June 2013, a majority of oncologists, primary care physicians, and other non-pain management or surgical specialists reported that REMS had a moderate or substantial impact on their interactions with patients [19]. Additionally, in our previous study on patient experiences with the same ETASU REMS as in the present study, we found that the programs made some patients more comfortable following their physicians’ advice [20].

The time and resources needed to meet requirements were a frequent point of discussion in the interviews. The FDA is currently partnering with the MITRE Corporation to develop “an open source proof-of-concept prototype” to better integrate REMS programs into existing health information systems and health care delivery processes, including electronic prescribing [21]. In addition to this initiative, the FDA can require programs to have online data entry portals instead of faxed or mailed submissions, while drug manufacturers can compensate physicians for reporting required clinical information. Such payment would have to be commensurate with effort so as not to create a perverse incentive to prescribe an ETASU REMS-covered drug.

Steps can also be taken to mitigate concerns raised by physicians about data privacy. These concerns mirrored those expressed by patients [14] and can be mitigated by requiring manufacturers to offer clearer explanations about what patient health information can be collected under ETASU REMS, for what purposes, and over what time. Direct FDA administration or required routine third-party audits of these programs might help reassure physicians that the data collected is being properly used for purposes such as pharmacovigilance.

Limitations of our study include physician participants from just 4 ETASU REMS. However, these programs shared core elements with other programs. Second, most physicians interviewed were from Massachusetts and academic medical centers. Third, our use of patient support groups might have resulted in skewed recruitment of specialists. Their views and experiences therefore may not have been representative of all physicians who prescribe these drugs. Work is currently underway to understand a broader range of physician experiences with REMS programs [22].

Our findings suggest that physician knowledge about ETASU REMS was strong. For some physicians, the programs provided reassurance and facilitated discussions about treatment. However, reforms are needed to ensure more efficient implementation and better protection of patient health information.

## Supporting information

S1 File (DOCX)
