# Supplementary material for: Physician experiences with and perceptions of risk evaluation and mitigation strategy programs with elements to assure safe use
Source: PLoS One. 2023 Jul 6;18(7):e0288008. doi: 10.1371/journal.pone.0288008 (PMC10325060; doi:10.1371/journal.pone.0288008)
Supplement: S1 File — (DOCX) [file pone.0288008.s001.docx]

**Physician Experiences with and Perceptions of**

**Risk Evaluation and Mitigation Strategy Programs with Elements to Assure Safe Use**

**Supplementary Appendix**

**SA-1: Interview Guide**

**I. INTRODUCTION/B­­ACKGROUND** (10 minutes)

Good [morning/afternoon/evening]. May I speak with [Dr. __________]? Hi, my name is [your name], and I am calling as part of a study on a medication safety program being conducted by researchers at Harvard Medical School and Brigham and Women’s Hospital in which you agreed to participate. This study is funded by the Greenwall Foundation, a non-profit bioethics organization. If you complete this interview, we will send you or a charity of your choice $150 in recognition of your time and effort. Is this still a good time for us to talk?

**IF NO, ARRANGE A TIME TO CALL BACK.**

To begin, let me confirm that you have prescribed [drug] in the past year. Is that right? [Ask any additional qualifying questions for the REMS (i.e., female of reproductive potential)]

**IF NO, THANK POTENTIAL RESPONDENT FOR (HIS/HER) TIME AND END THE INTERVIEW. IF YES, CONTINUE.**

Thanks. Just to clarify, if there is a question that you would rather not answer, we can skip it. This interview is being recorded so that our team can faithfully capture your responses, which we hope to use to determine how the safety program can be effectively and fairly structured. Let’s begin.

**Demographic Data**

1. Can you please describe your **practice setting**?
2. Are you a **specialist** for which [drug] is indicated?
3. How **long** have you been **in practice**?
4. How **frequently** have you **prescribed** the drug? For new prescriptions and refills?
5. How much staffing support is there in your practice? How busy is the practice?

**II. UTILITY AND BURDEN** (30 minutes)

Background learning method and understanding

From what sources do you usually learn about drug benefits and risks?

Which sources of information do you prefer and why?

PROBE

How often do you check for updates on the drugs you prescribe?

Do you learn about drug benefits and risks directly from pharmaceutical representatives/detailers?

**IF YES**: Can you tell me more about this interaction?

**IF NO**: Why do you refrain from getting information for this source?

As mentioned previously, [drug] is subject to a special safety program, risk evaluation and mitigation strategies with elements to assure safe use—otherwise known as an ETASU REMS. Are you aware of these programs generally?

PROBE: Based on your understanding, when can such programs be imposed? When should they be imposed?

SEGUE**:** ETASU REMS are programs to manage potential serious risks associated with a medicine. They may include person, place, and time restrictions on dispensing or administration as well as mandatory registry enrollment and follow-up testing, among other requirements. Since drugs and their associated risks can be very different from each other, each ETASU REMS is also different.

Questions about safety program

1. The FDA says that [drug] has a safety concern that warrants an ETASU REMS program. To what extent did the safety program impact your willingness to prescribe [drug]? Why or why not?

PROBE: Can you tell me more about how you specifically weigh the benefits and risks of prescribing [drug]?

1. What can you recall about the safety program?

[Refer to **FAST FACTS** to remind interviewee about the core components of the safety program]

1. What do you think about [drug’s] safety program?
   PROBE: Did you find the safety program helpful or not? Why?
2. Do you recall signing an enrollment/agreement form before being able to prescribe [drug]?

**IF NO TO QUESTION 7, PROCEED TO PROBE IF PHYSICIAN FORMS EXIST; OTHERWISE PROCEED TO QUESTION 10**

1. Can you recall if there were specific terms that you agreed to follow?
   1. **IF YES**: What were they?

PROBE: [Refer to **PHYSICIAN ENROLLMENT FORM]**

1. What did you think about the enrollment/agreement form?

PROBE

1. What effect, if any, did the form have on your patient care?
2. Can you give your thoughts on requiring such forms?
3. Was your patient required to sign an enrollment/agreement form?

**IF NO**

- 1. Would knowing that your patient was required to sign an enrollment/agreement form have changed how you felt about prescribing [drug]? Why or why not?

**IF YES**

[Refer to **PATIENT ENROLLMENT FORM**]

- 1. How did you feel about that?
  2. In your view, how did patients respond to the form?

PROBE: What impact, if any, did the form have on patients’ perception of the risk associated with taking the drug?

1. As part of the safety program, were you asked to answer questions or take a quiz to test your knowledge about the drug or the safety program?
   1. **IF YES**: What did you think about these questions?

PROBE: Were the questions helpful or not?

- 1. **IF NO**: What are your thoughts on requiring testing/questions?

1. Does the REMS program require you to report adverse events to the manufacturer? If so, what have your experiences been with this process?
2. Have your patients recounted any problems filling/receiving their prescription for [drug]? If yes, what problems?

PROBE

1. Have any particular groups been more heavily affected? If so, how?
2. What, if any, issues have patients reported concerning their insurance?
3. Have you encountered any problems with the safety program for [drug]? If so, what problems and were they resolved?

PROBE: Can you describe the level of work involved in complying with the REMS?

1. Given your experience with the safety program for [drug], how, if at all, do you think it could be improved?

PROBE

[Components: patient enrollment, provider enrollment, testing/evaluation, distribution, company access to information, adverse event reporting]

1. Are there any components would you eliminate? keep? change?
2. Who do you ideally think should run a REMS program? What are the potential advantages/ disadvantages of such a setup?
3. If you were designing a safety program for [drug] from scratch, what would be its main features given your knowledge of the risks [drug] poses?

**III. PRIVACY** (15 minutes)

Now, I’d like to discuss the issue of professional and health privacy.

1. You answered that you [were/were not] required to submit enrollment/agreement forms to the drug company. Have you been asked to provide other information to the drug company as part of the safety program? If so, what?

**IF NO TO QUESTION 17, PROCEED TO QUESTION 20 or 21**

**IF YES TO QUESTION 17, PROCEED TO QUESTION 18**

1. If so, what kind of information?

PROBE: Has the drug company ever asked you to forward them the personal health information for a patient taking [drug]?

1. Have you ever been offered payment to forward this information?
2. **IF ACCESS TO PERSONAL HEALTH INFORMATION REQUIRED**: The terms of the safety program state [read company access statement]. What do you think about this provision?

PROBE

- 1. How do you feel about drug companies conditioning receipt of medication upon access to personal health information? Are there any conditions you would like to see imposed on such access? If so, what?

1. **IF ACCESS TO PERSONAL HEALTH INFORMATION NOT REQUIRED**: Some safety programs require drug company access to personal health information. Would that be okay? Are there any conditions you would like to see imposed on such access? If so, what?

That’s all of the questions I have for you. Is there anything I haven’t asked about that you would like to discuss?

I will be in touch soon regarding compensation. Would you like to provide the address that we can use to send your gift card now or do you prefer to follow up with this information via email?

If additional questions arise, please contact the study principal investigator, XX at XX.

Thank you for your time and your insights. I appreciate you speaking with me.

**SA-2: Coding Tree**

Utility

Risk-Benefit Tradeoffs

Data Collection

Characteristics of Physician

Practical Consequences for Physicians

Practical Consequences for Patients

Physician Understanding of REMS

Education

Why and When REMS Are Applied to Drugs

Utility of REMS

REMS Improvements

Highly Specialized vs. Less Specialized Physicians

Data Sharing and Confidentiality

Perceptions of Risk

Role of Manufacturers

Access to Data and Support

Knowledge or Recollection

Reporting of Adverse Events

Perceptions of Drug Risks

Risks and Benefits of Medication

Burden

Cost, Insurance, and Access

Education

Practice Setting

Learning About Drugs

Administrative Burden or Problem

Effect on Practice
